# Inhibition of p38 ameliorates axonal loss with modulation of autophagy in TNF-induced optic nerve damage

**DOI:** 10.1007/s10792-023-02706-1

**Published:** 2023-04-16

**Authors:** Kana Sase, Chihiro Tsukahara, Naoki Fujita, Ibuki Arizono, Mizuki Otsubo, Yasushi Kitaoka

**Affiliations:** 1 Department of Ophthalmology, St. Marianna University School of Medicine, 2-16-1 Sugao, Miyamae-ku, Kawasaki, Kanagawa 216-8511 Japan; 2 Department of Molecular Neuroscience, St. Marianna University Graduate School of Medicine, Kawasaki, Japan

**Keywords:** p38, Autophagy, p62, LC3, TNF

## Abstract

**Purpose:**

A relationship between p38 and autophagy remains debated. The aim of the current study is to investigate whether an inhibitor of p38 prevents axon loss induced by TNF and whether it affects autophagy.

**Methods:**

Rats were given intravitreal injection of TNF, TNF plus SB203580, a p38 inhibitor, or SB203580 alone. Immunoblot analysis was performed to examine p62 expression which is a marker of autophagic flux and LC3-II expression which is an autophagy marker in optic nerves 1 week after intravitreal injection. Morphometric analysis of axons was performed to evaluate the effects of SB203580 against TNF-induced optic nerve damage 2 weeks after intravitreal injection. Immunohistochemical analysis was performed to evaluate the expressions of LC3, neurofilament, phosphorylated p38 and p62 in the optic nerve.

**Results:**

Quantification of axon number showed that TNF-induced axon loss was significantly protected by SB203580. Immunoblot analysis showed that the increase of p62 induced by TNF was totally eliminated by SB203580, and the SB203580 alone injection decreased the expression of p62. The level of LC3-II was significantly upregulated in the TNF plus SB203580 group compared with the TNF alone group, and the SB203580 alone injection increased the expression of LC3-II. Immunohistochemical analysis showed that LC3 immunoreactivity was found in the neurofilament positive fibers and that these immunoreactivities were enhanced by SB203580. Some colocalizations of p-p38 and p62 were observed in the TNF-treated optic nerve.

**Conclusion:**

These results suggest that inhibition of p38 exerts axonal protection with upregulated autophagy in TNF-induced optic nerve damage.

## Introduction

Autophagy is a cellular process that eliminates unnecessary proteins and subcellular elements through lysosome-related degradation to maintain homeostasis and be associated with differentiation, development and survival [1]. Autophagy has been associated with the pathophysiology of some human diseases, such as neurodegeneration, including glaucoma [2–5]. However, the role of autophagy in retinal ganglion cell (RGC) death and optic nerve degeneration remains controversial [6–10]. It was reported that dysregulation of autophagy contributes to neurodegenerations in glaucoma [11]. A recent study reported that the role of autophagy in RGCs during ocular hypertension development might differ in a time-dependent manner [12]. Our previous reports showed that autophagy induction leads to axonal protection in the TNF-induced optic nerve degenerative model [13, 14]. We recently reported that Akebia Saponin D (ASD), which is also known as an autophagy inducer exerts axonal protection in the TNF-induced optic nerve degeneration model [15]. That study also found that phosphorylated-p38 (p-p38) exists in optic nerve axons and is upregulated by TNF, and that this upregulation was prevented by ASD [15]. However, a recent study demonstrated that resveratrol, which is also known as an autophagy inducer ameliorated the high glucose-induced oxidative damage in human lens epithelial cells by promoting autophagy through the upregulation of p-p38 [16]. Thus, whether activation or inhibition of p38 may lead to autophagy activation remains debated. To address this question, the current study used an inhibitor of p38 and evaluated autophagy status with axonal histological conditions.

## Materials and Methods

### Animals

Eight-week-old male Wistar rats were used in this experiment. This experiment was approved by the Ethics Committee of the Institute of Experimental Animals of St. Marianna University Graduate School of Medicine. This study was performed according to the ARVO statement for the Use of Animals in Ophthalmic and Vision Research. The rats were housed in controlled conditions (23 ± 1 °C, humidity at 55 ± 5%, and light from 06:00 to 18:00).

### Intravitreal injection

Intravitreal injection was performed as described previously [14]. In anesthetized rats with a combination of ketamine and xylazine, a single 2-µl injection of 10 ng TNF was administered intravitreally to the right eye. For the SB203580 (Santa Cruz) treatment, simultaneous injection of 2 nmol of SB203580 and 10 ng of TNF was performed intravitreally. SB203580 alone group was also tested. TNF and SB203580 were dissolved in dimethylsulfoxide (DMSO; EMD Millipore Corp) and diluted with phosphate-buffered saline (PBS). The same amount of DMSO in PBS was injected as a control. One or 2 weeks after the intravitreal injections, the rats were euthanized with an intraperitoneal overdose of sodium pentobarbital.

### Quantification of axon number

Axon number counting was performed in optic nerves from 16 rats as described previously [14, 17]. The optic nerves were collected 2 weeks after intravitreal administration. They were fixed by immersion in Karnovsky’s solution and embedded in acrylic resin. Cross sections were made starting 1 mm from the eyeball and stained with 1% paraphenylen-diamine (Sigma-Aldrich) in methanol. Five images (center and periphery in quadrant; 5850 µm^2^ each; total area of 29,250 µm^2^ per eye) were acquired and quantified using an image processing software (Aphelion).

### Immunoblot analysis

One week after intravitreal injection, optic nerves (4 mm in length) were collected, homogenized, and centrifuged at 15,000 × *g* for 15 min at 4 °C as described previously [15]. Protein concentrations in supernatants were measured. Samples (3 µg per lane) were applied to SDS-PAGE gels and transferred to PVDF membranes. After blocking, membranes were first reacted with p62 antibody (1:200; MBL), LC3 antibody (1:200; MBL), or anti-β-actin antibody (1:500; Sigma-Aldrich) in Tris buffered saline. Membranes were next reacted to secondary antibodies; peroxidase-labeled anti-rabbit IgG antibody (1: 5000; Cappel, Solon, OH, USA) or peroxidase-labeled anti-mouse IgG antibody. Immunoblotting was visualized with an ECL detection system.

### Immunohistochemistry

The eyes were enucleated 1 week after intravitreal administration, immersed in 4% paraformaldehyde, processed, and embedded in paraffin. Transverse sections were made through the optic disc and blocked with 1% bovine serum. The primary antibodies were anti-LC3 antibody (1:100; MBL), anti-neurofilament-L antibody (a marker of neurons; 1:100; DAKO), p62 antibody (1:100; Sigma-Aldrich), and anti-p-p38 antibody (1:100; Cell Signaling). FITC-labeled and rhodamine-labeled antibodies were diluted 1:5000 and used as secondary antibodies. The images were captured with a confocal microscopy system (Zen; Carl Zeiss QEC GmbH).

### Statistical analysis

Differences among groups were analyzed using one-way ANOVA, with post-hoc Tukey’s HSD test. The results were considered statistically significant when probability value of less than 0.05. Statistical significance is reported as asterisks in graphs (**** for *P* < 0.0001, *** for *P* < 0.0005, ** for *P* < 0.01, * for* P* < 0.05).

## Results

### Effects of p38 inhibition against TNF-induced axon loss

**Fig. 1:**
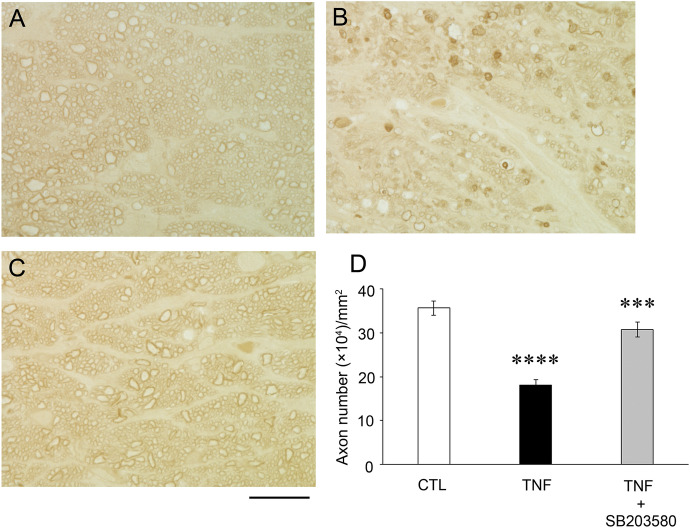
SB203580 prevents axon loss in the TNF-induced optic nerve damage. PPD-stained axons at 2 weeks after **A** vehicle, **B** TNF, **C** TNF + 2 nmol SB203580 administration. Scale bar = 10 µm; magnification, × 100. **D** Quantification of axon numbers. n = 5–10 per group. ****P < 0.0001 compared with CTL; ***P < 0.0005 compared with TNF

Consistent with our previous studies [14, 15] and compared with the control group (Fig. 1a), the TNF group showed apparent degenerative findings in the optic nerve (Fig. 1b). However, the TNF plus SB203580 group showed obvious protective findings compared with the TNF alone group. (Fig. 1c). Morphometric analysis showed that there was a significant difference between the control group and TNF group (Fig. 1d). In addition, there was a significant difference between the TNF group and TNF plus SB203580 group (Fig. 1d).

### Effects of TNF and p38 inhibition on p62 protein levels

**Fig. 2:**
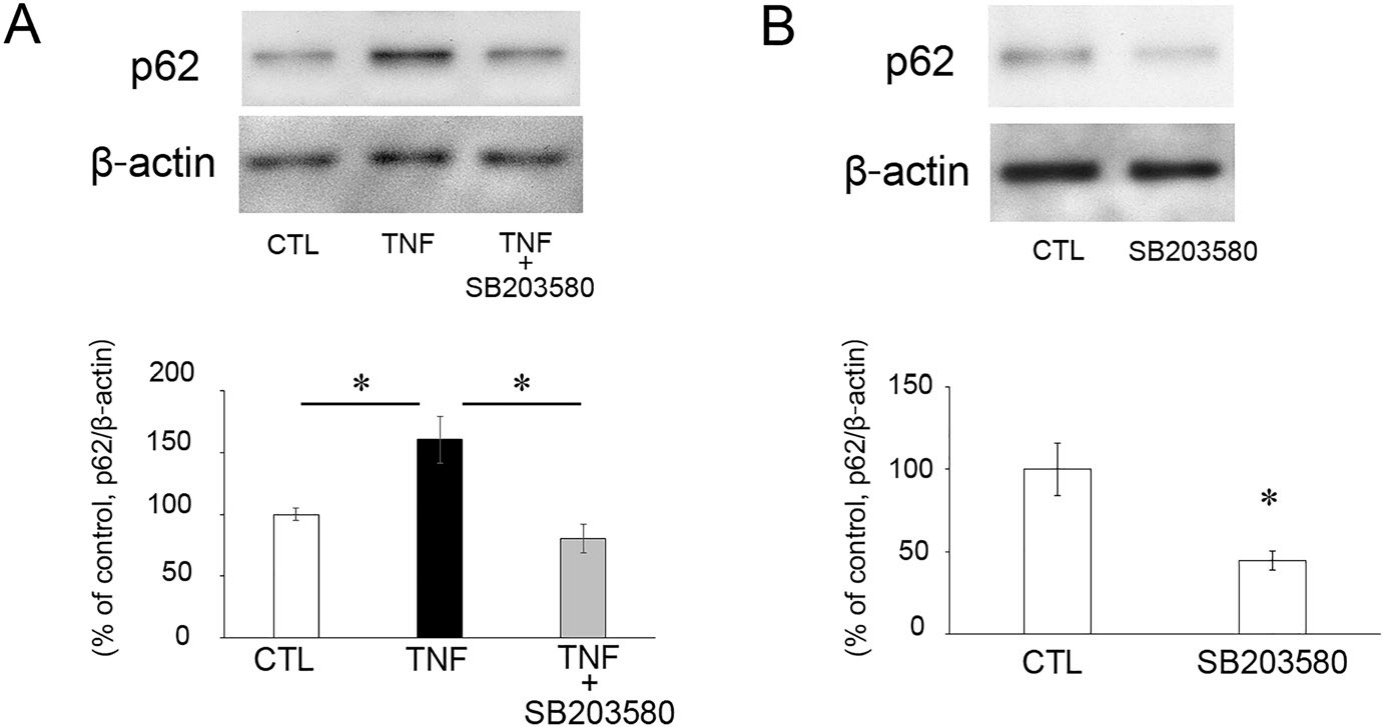
Expression of p62 protein in the optic nerves at 1 week after intravitreal administration of vehicle, TNF, or TNF + 2 nmol SB203580 (**A**) and vehicle or 2 nmol SB203580 (**B**). Normalization was conducted using β-actin levels in the same sample. n = 3–4 per group. *P < 0.05

To examine the effects of SB203580 on autophagic status, we evaluated the changes in protein levels of p62, a maker of autophagy flux. Consistent with our previous studies [14, 15], TNF injection significantly increased p62 protein level (Fig. 2a). The increased p62 expression induced by TNF was completely suppressed by SB203580 (Fig. 2a). Additionally, the SB203580 alone injection decreased the expression of p62 (Fig. 2b).

### Effects of TNF and p38 inhibition on LC3-II protein levels

**Fig. 3:**
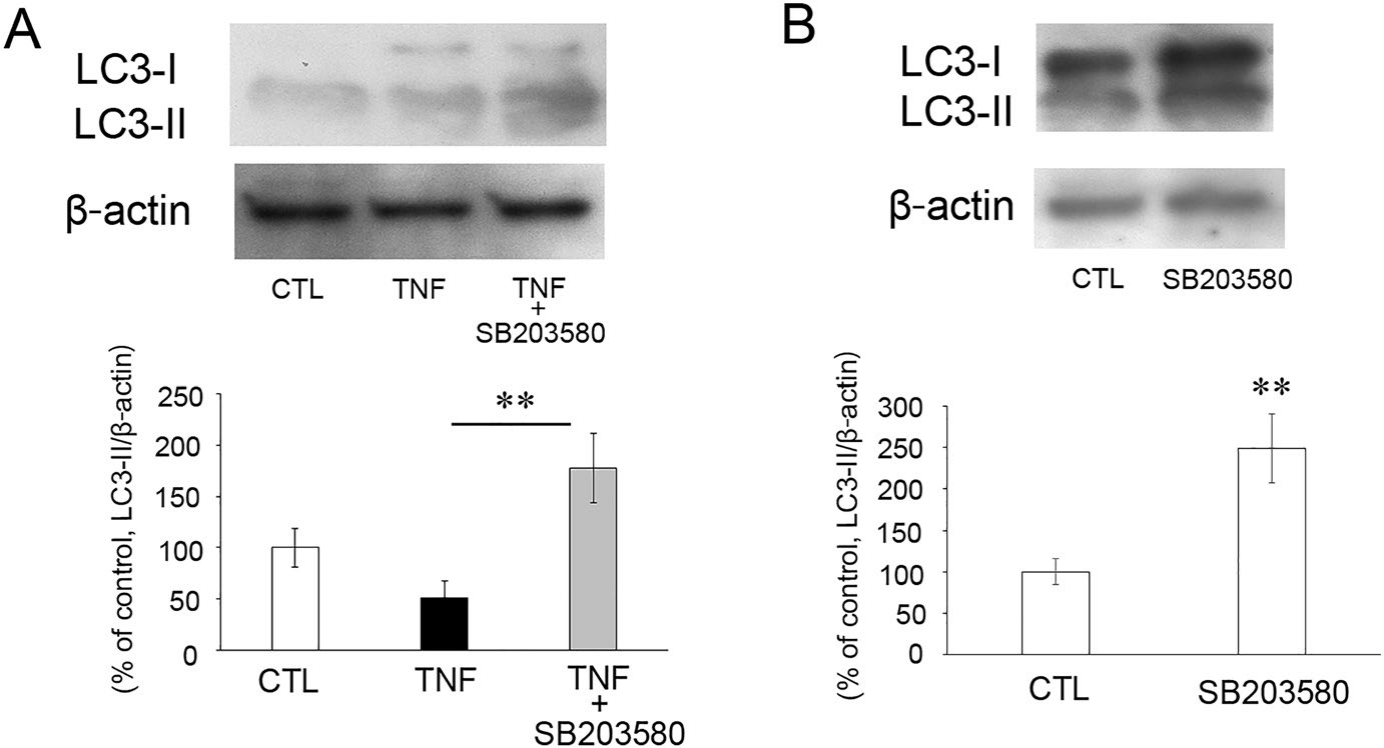
Expression of LC3-II protein in the optic nerves at 1 week after intravitreal administration of vehicle, TNF, or TNF + 2 nmol SB203580 (**A**) and vehicle or 2 nmol SB203580 (**B**). Normalization was conducted using β-actin levels in the same sample. n = 6–8 per group. **P < 0.01

We evaluated the effect of SB203580 on the level of LC3-II, an autophagic marker. Consistent with our previous studies [14, 15], the expression of LC3-II levels did not statistically change in the TNF group compared with the control group (Fig. 3a). However, the level of LC3-II was significantly upregulated in the TNF plus SB203580 group compared with the TNF alone group (Fig. 3a). Additionally, the SB203580 alone injection increased the expression of LC3-II (Fig. 3b).

### LC3 immunoreactivity in optic nerve

**Fig. 4:**
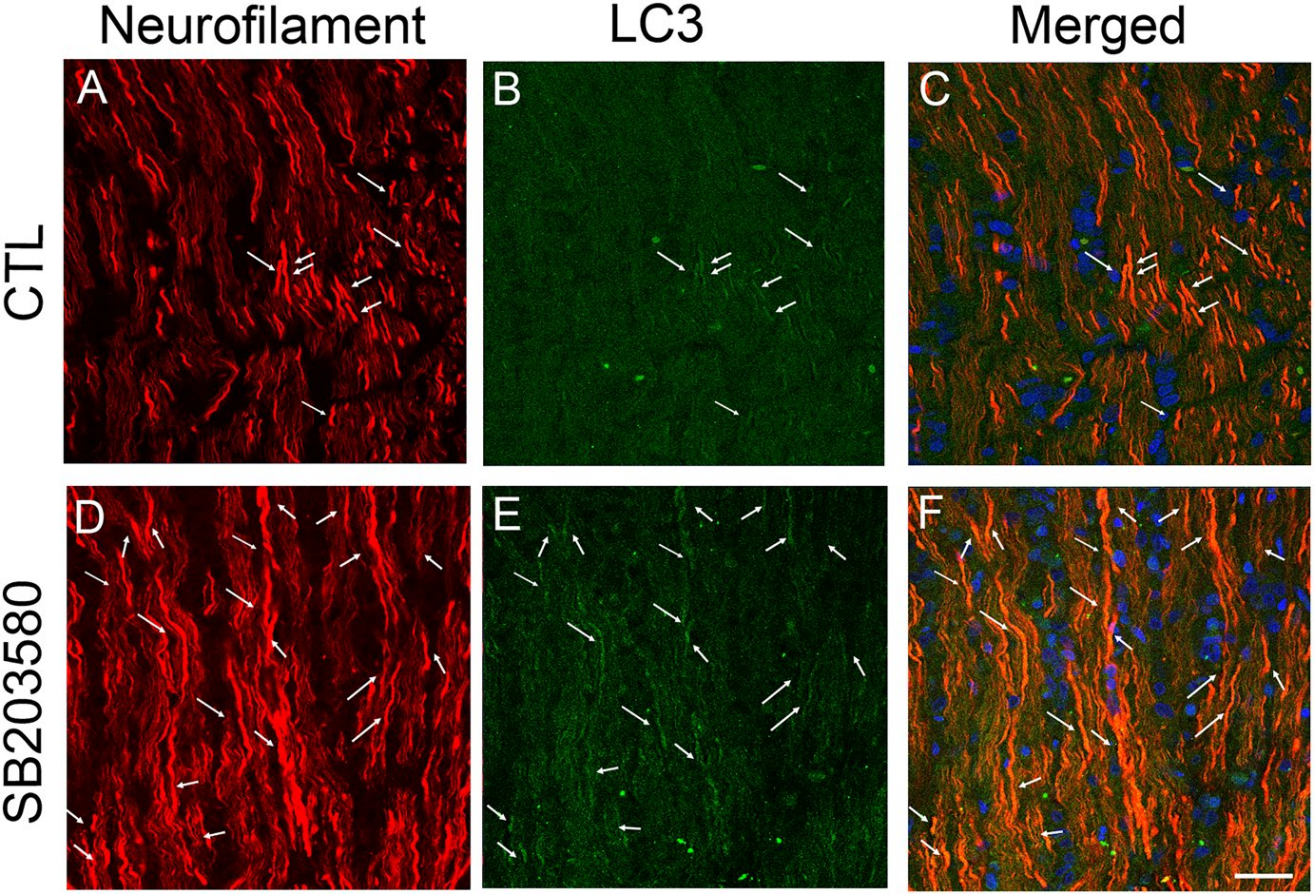
Immunohistochemical analysis of the optic nerve. Double staining of LC3 and neurofilament showed some colocalizations in the control group (**A**–**C**). In the SB203580 group (**D**–**F**), expression of LC3 seemed to be more abundant compared with the control group. Arrows indicate colocalizations. Scale bar = 50 µm

**Fig. 5:**
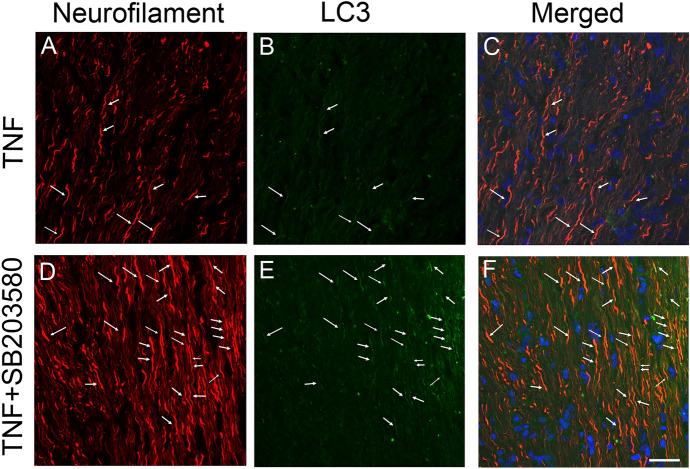
Immunohistochemical analysis of the optic nerve. Double staining of LC3 and neurofilament showed some colocalizations in the TNF group (**A**–**C**). In the SB203580 plus TNF group (**D**–**F**), expression of LC3 seemed to be more abundant compared with the TNF group. Arrows indicate colocalizations. Scale bar = 50 µm

Since we previously observed that LC3-immunopositive dots were located inside neurofilament-positive fibers [14], the current study tested the effect of SB203580 on these immunoreactivities. Some colocalizations of LC3 and neurofilament were seen in the control group (Fig. 4a–c). Similar to the Western blot findings, LC3 immunoreactivity was enhanced in the SB203580 group compared with the control group, and some colocalizations were observed (Fig. 4d–f). In addition, SB203580 treatment also enhanced LC3 immunoreactivity in the TNF group, and some colocalizations were observed (Fig. 5).

### p62 and p-p38 immunoreactivities in optic nerve

**Fig. 6:**
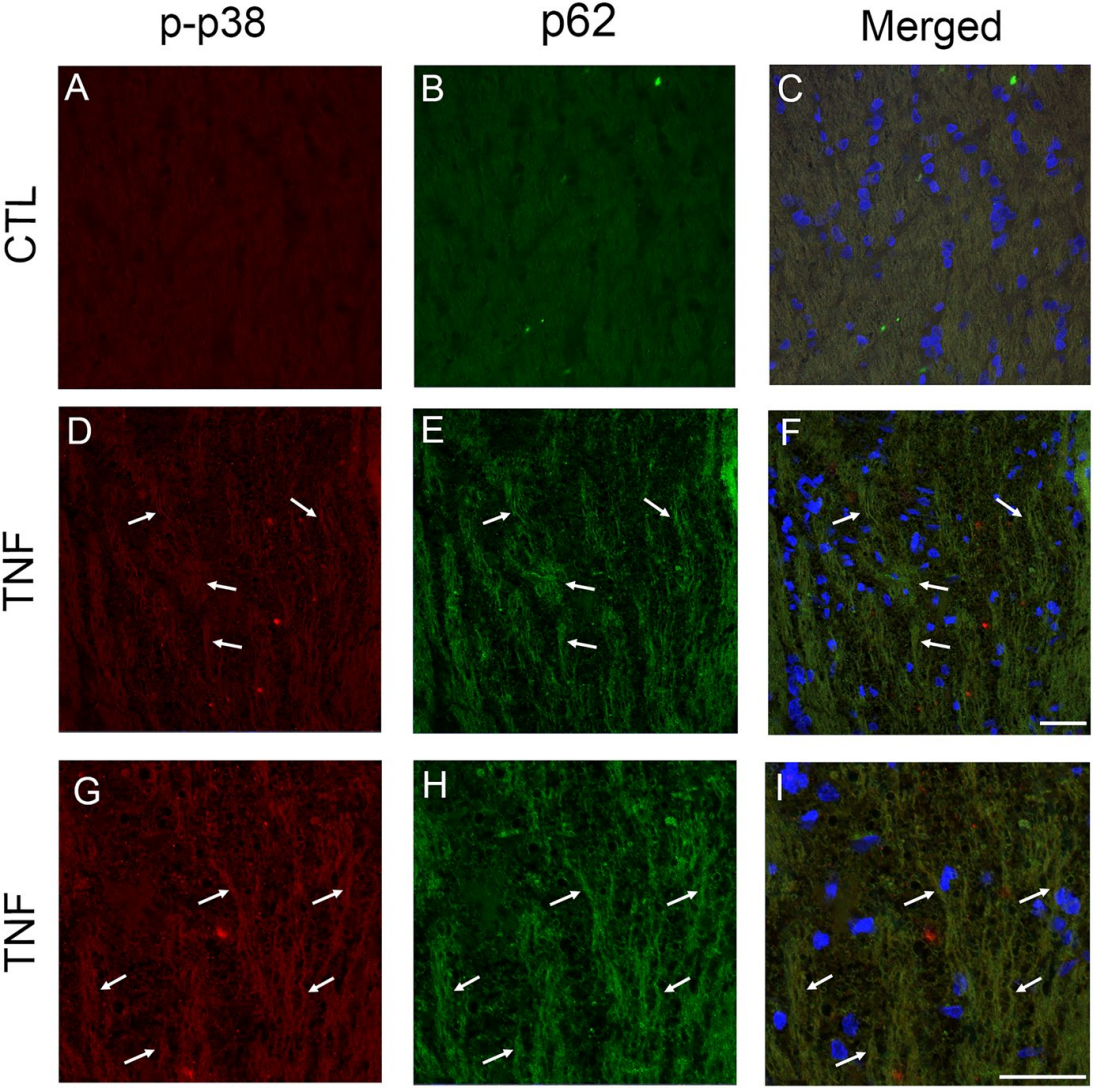
Immunohistochemical analysis of the optic nerve. Double staining of p-p38 and p62 showed some colocalizations in the TNF group (**D**–**I**) compared with the control group (**A**–**C**). High magnification images also showed some colocalizations (**G**–**I**). Arrows indicate colocalizations. Scale bar = 50 µm

We previously observed that p-p38 immunoreactivity was colocalized with neurofilament-positive fibers in the TNF model [15]. The current study examined the localizations of p62 and p-p38 in optic nerve. Some colocalizations of p62 and p-p38 were found in the TNF group (Fig. 6d–i). Similar to the Western blot findings, p62 and p-p38 immunoreactivities were enhanced in the TNF group compared with the control group (Fig. 6).

## Discussion

In addition to the previous study showing that SB203580 protected RGC death induced by two different models such as optic nerve injury and NMDA administration [18, 19], the present study evaluated its effect on axons in the other model. The current study found that SB203580, a p38 inhibitor, exerts significant protective effect on axon loss in the TNF-induced optic nerve degeneration. Consistent with this axonal protection, a previous study reported that topical eye delivery of p38 inhibitor Ro3206145 protects RGC axons following microbeads injection ocular hypertension model in rats [20]. Same group recently demonstrated that topical eye delivery of p38 inhibitor BIRB796 protects RGC axons in this ocular hypertension model [21]. On the other hand, in primary cultured cortical neurons, a significant decrease in neurofilament protein level induced by interleukin (IL)-1β was ameliorated by SB203580, suggesting that IL-1β-induced axon loss was protected by p38 inhibition [22]. These findings suggest that SB203580 can not only protect neuronal cells but also their axons.

Autophagy is a dynamic process and the assessments of LC3-II and p62 are informative. Inhibition of autophagy has been linked to the augmentation of p62 and its decline is associated with autophagy flux enhancement [23]. Our recent study found that the levels of p-p38 and p62 were elevated by TNF in the optic nerve, and these elevations were suppressed by ASD, an autophagy inducer [15]. Our current immunohistochemical study found some colocalizations of p-p38 and p62 in the TNF-treated optic nerve. This is in line with a previous study demonstrating colocalization of p-p38 and p62 in the HeLa cells [24]. Although the peak of p-p38 upregulation was 1 week after TNF injection, upregulations of p62 were observed at both 1 and 2 weeks, implying that p-p38 exists upstream of p62 [15]. Thus, the present study tested this point using p38 inhibitor SB203580. In the current study, the increase of p62 induced by TNF was abolished by SB203580 and it alone treatment significantly diminished p62 levels, suggesting that inhibition of p38 may enhance autophagy flux in the optic nerve. It is noteworthy that SB203580 may restore impaired autophagy flux in a subarachnoid hemorrhage model [25]. Moreover, we found that the levels of LC3-II in both the TNF group and the control group were significantly augmented by SB203580. Furthermore, the current immunohistochemical analysis revealed that SB203580 augmented LC3 immunoreactivity in both the control group and the TNF group. Consistently, a previous study showed that p38 knockdown increased LC3-II levels in the mouse brain [26]. That study also showed that SB203580 treatment significantly increased the autophagosome numbers in the SH-SY5Y cells [26], demonstrating that deletion of p38 can enhance autophagy in neuron both in vivo and in vitro. It is interesting to note that SB203580 reduced p62 level and upregulated LC3-II level in the hippocampal neurons in chronic intermittent hypoxia model rats [27]. That study also demonstrated that SB203580 increased autophagic vacuole numbers and protected hippocampal CA1 area neurons [27]. Therefore, it is reasonable to suggest that p38 exists upstream of p62 and that inhibition of p38 can promote autophagy, thereby leading to neuroprotection. It is interesting to consider how p38 inhibition with autophagy modulation can be used as a neuroprotective therapy. There is a possibility that above mentioned eye drop of p38 inhibitor may modulate autophagy, but further study will be necessary to elucidate this point.

## Data Availability

The datasets used and/or analyzed during the present study are available from the corresponding author on reasonable request.
